# Effects of major urban redesign on sedentary behavior, physical activity, active transport and health-related quality of life in adults

**DOI:** 10.1186/s12889-023-16035-6

**Published:** 2023-06-15

**Authors:** N. E.H. Stappers, M. P.M. Bekker, M. W.J. Jansen, S. P.J. Kremers, N. K. de Vries, J. Schipperijn, D. H.H. Van Kann

**Affiliations:** 1 NUTRIM School of Nutrition and Translational Research in Metabolism, Department of Health Promotion, Maastricht University, Debyeplein 1, Maastricht, 6229HA The Netherlands; 2 Center for Space, Place and Society, Social Sciences Group, Wageningen University, Wageningen, The Netherlands; 3 CAPHRI Care and Public Health Research Institute, Department of Health Services Research, Maastricht University, Maastricht, The Netherlands; 4 Academic Collaborative Center for Public Health, Public Health Service South-Limburg, Heerlen, The Netherlands; 5 CAPHRI Care and Public Health Research Institute, Department of Health Promotion, Maastricht University, Maastricht, The Netherlands; 6 Research Unit for Active Living, Department of Sports Science and Clinical Biomechanics, University of Southern Denmark, Odense, Denmark; 7 School of Sport Studies, Fontys University of Applied Sciences, Eindhoven, The Netherlands

**Keywords:** Infrastructural change, Physical activity, Active transport, Health-related quality of life

## Abstract

**Background:**

The built environment is increasingly recognized as a determinant for health and health behaviors. Existing evidence regarding the relationship between environment and health (behaviors) is varying in significance and magnitude, and more high-quality longitudinal studies are needed. The aim of this study was to evaluate the effects of a major urban redesign project on physical activity (PA), sedentary behavior (SB), active transport (AT), health-related quality of life (HRQOL), social activities (SA) and meaningfulness, at 29–39 months after opening of the reconstructed area.

**Methods:**

PA and AT were measured using accelerometers and GPS loggers. HRQOL and sociodemographic characteristics were assessed using questionnaires. In total, 241 participants provided valid data at baseline and follow-up. We distinguished three groups, based on proximity to the intervention area: maximal exposure group, minimal exposure group and no exposure group.

**Results:**

Both the maximal and minimal exposure groups showed significantly different trends regarding transport-based PA levels compared to the no exposure group. In the exposure groups SB decreased, while it increased in the no exposure group. Also, transport-based light intensity PA remained stable in the exposure groups, while it significantly decreased in the no exposure group. No intervention effects were found for total daily PA levels. Scores on SA and meaningfulness increased in the maximal exposure group and decreased in the minimal and no exposure group, but changes were not statistically significant.

**Conclusion:**

The results of this study emphasize the potential of the built environment in changing SB and highlights the relevance of longer-term follow-up measurements to explore the full potential of urban redesign projects.

**Trial registration:**

This research was retrospectively registered at the Netherlands Trial Register (NL8108).

**Supplementary Information:**

The online version contains supplementary material available at 10.1186/s12889-023-16035-6.

## Background

Over the past decades, the built environment is increasingly recognized as a determinant of health and health behaviors. Several socioecological models explain how changes in the environment can lead to improved health at the individual-level through changes in the organizational, intrapersonal and interpersonal level of influence [1–3]. Systematic reviews show that the built environment can affect health behaviors such as physical activity, active transport and healthy eating, both in the entire population [4, 5], and in subgroups in society [6, 7]. However, the effects are varying in significance and magnitude [8, 9].

Physical activity is considered to be one of many pathways between the environment and health and well-being [10]. Other pathways that were identified were for example community interaction, healthy eating, social relationships, leisure and work [11]. Also, air quality is found to be significantly correlated with quality of life and life satisfaction [12, 13]. An extensive amount of research concerning the relationship between the environment and health assesses the effect of green space on general and mental health [14, 15]. Most studies indicate that there is a beneficial relationship between green space and health, but the evidence is weak [14]. This was confirmed by a review that evaluated the effects of improving green infrastructure and urban regeneration on mental health and well-being in adults, which only found weak evidence for the relationship between the built environment and quality-of-life [16].

The inconclusive results of previous studies are due to several factors. For research on the relationship between environment and physical activity, one of the main shortcomings of existing research is the relatively short follow-up term [4, 9]. While in many natural experimental studies the time between exposure and follow-up is less than 24 months, behavioral change might take more than 3 years to actually occur [17]. Especially in large projects, several external factors can influence follow-up times, for example delays in implementation of urban redesign plans or the typical short duration of research contracts and projects [18]. This lack of longer-term follow-up studies is unfortunate, especially since in some interventions, evidence may accumulate over time to show the strength of their outcomes [19]. For research regarding the relationship between the built environment and general health and wellbeing, a large number of existing studies are based on cross-sectional analyses, which makes it difficult to explore causal relationships, and the risk of bias was considered to be serious in the majority of the studies [16]. Lastly, a recent review of reviews concluded that future research should focus on improving study quality, for example by using longitudinal methods and novel technologies such as GPS- data and ecological momentary assessments [20].

An opportunity to design a high-quality longitudinal natural experiment assessing the effects of the built environment on both physical activity levels and health-related quality of life presented itself with a major urban highway redesign project running through the Dutch city of Maastricht (a city in The Netherlands). This longitudinal natural experiment lasted for six years, of which a first follow-up measurement took place between 3 and 15 months after the opening of the new infrastructure. The results of this study showed no increases in total or transport-based physical activity levels in inhabitants of the intervention area, but we found indications that the infrastructural change might prevent the increase in transport-based sedentary behavior over time [21]. The last follow-up measurement took place 29–39 months after the official opening of the new tunnel infrastructure in December 2016. Hereby, the current study aims to evaluate the longer-term effects of a major infrastructural redesign project on the physical activity levels and self-reported health-related quality of life of adults. To our knowledge, this is one of the first large-scale longitudinal studies that used device-based measurements and a follow-up time of at least 2 years after opening of the newly designed area.

## Methods

### Green carpet

In 2016, a crosstown highway was tunneled and the space on top of this tunnel was redesigned and included new infrastructure, houses and commercial spaces [22]. The new infrastructure has a length of 2.3 km and consists of a semi-paved middle path prioritized for pedestrians, bicyclists and recreation, accompanied by one-way streets for slow local traffic at both sides of the path. The middle path is separated from the one-way streets by trees and greenery, creating the so-called Green Carpet (www.mijngroeneloper.nl). The Green Carpet was officially opened in April 2018 and the construction of houses and commercial spaces started right after but is still ongoing until 2026.

### Design of the experiment

The design of this study is a natural experiment with three exposure groups, based on the proximity to the newly constructed area. As previously described in Stappers et al. (2022), the participants in the maximal exposure group had the closest proximity to the intervention area (approximately between 0 and 2000 m from intervention area), as they were living in a neighborhood directly bordering the Green Carpet [21]. The minimal exposure group consisted of individuals living in other parts of the same city but further away from the intervention area (approximately between 2500 and 5000 m from intervention area), so they were expected to be less exposed to the intervention area. Participants in the no exposure group lived in a different city in the same region (> 20 km from intervention area) and were not expected to be exposed to the newly designed area. A map with the geographical location of the exposure groups and intervention area can be found in the supplementary materials.

The average age distribution, percentage of social housing, population density and socioeconomic status of the neighborhoods in each exposure group are presented in Table 1. The age distribution in the entire population of the maximal, minimal and no exposure groups, are comparable, but the maximal exposure group has slightly more younger (< 25 years) inhabitants, and the minimal exposure group slightly more older (> 65 years) inhabitants. The percentage houses that are marked as social housing ranges between 33 and 36%. Population density was highest in the no exposure group and lowest in the maximal exposure group. Also, the socioeconomic status of the included neighborhoods was lower than the national average for all three exposure groups (Table 1).

**Table 1:** Characteristics of neighborhoods in the minimal, maximal and no exposure groups

	Maximal exposure	Minimal exposure	No exposure
Age distribution			
< 25 y	29%	27%	24%
25–65 y	51%	48%	55%
65 + y	20%	25%	21%
Percentage of social housing	36%	33%	33%
Population density (inhabitants/km2)	1675,5	1950,7	3663,0
Socioeconomic status*	-0,131	-0,200	-0,271

* Standardized score based on financial situation, educational level and workstatus. Score reflects the difference from the average neighborhood in the Netherlands. Source: Netherlands Statistics

Participants were adults (≥ 18 years) who are able to walk without walking aids, and able to fill out a Dutch questionnaire (with or without help). Recruitment was done via various on- and offline channels, such as social media, posters, flyers at supermarkets, key figures in the selected neighborhoods, advertisements in local and regional newspapers, and personalized mailing to a random sample of the inhabitants of the cities of Maastricht and Heerlen.

**Fig. 1:**
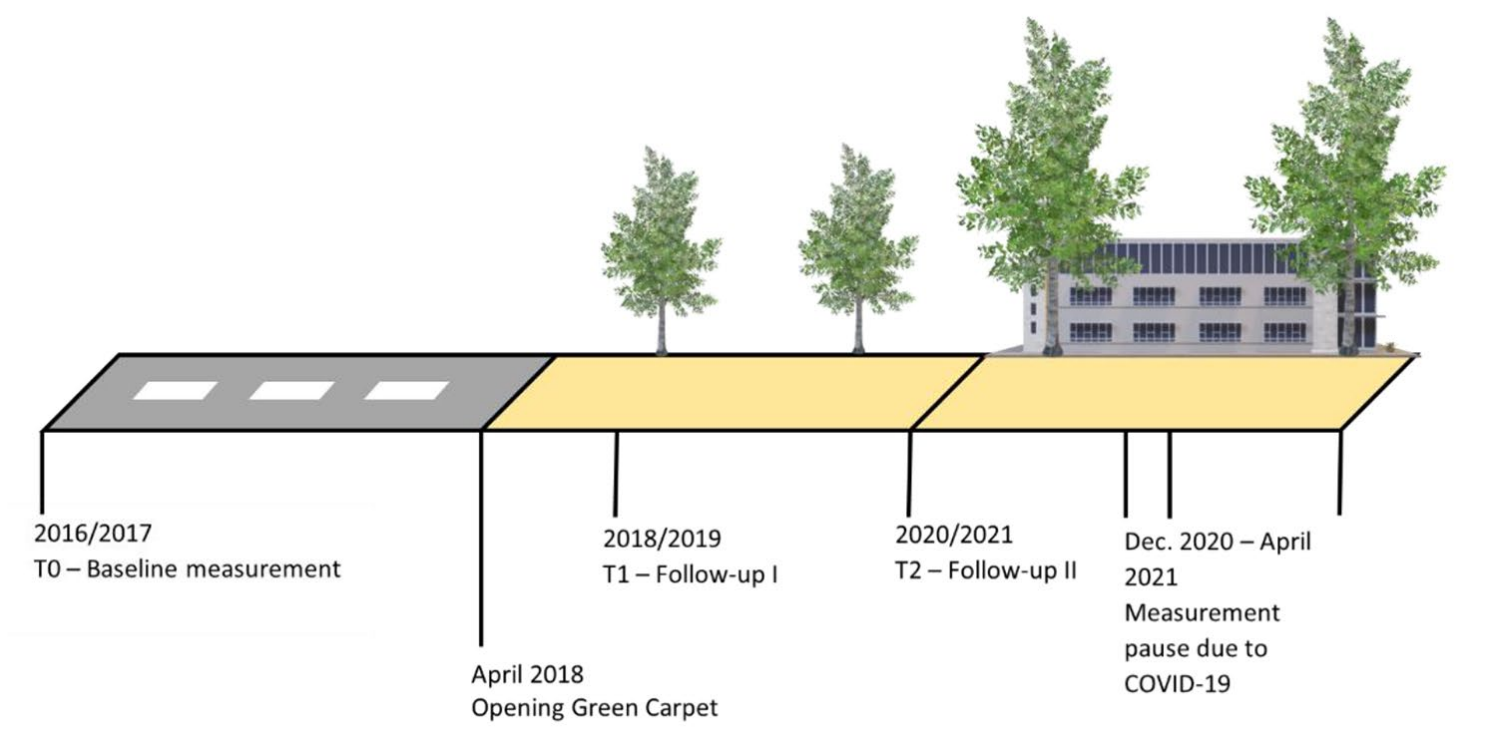
Timeline of natural experiment

All participants were measured at three points in time: baseline (T0; July 2016 – July 2017), follow-up I (T1; July 2018 – July 2019) and follow-up II (T2; September 2020 – July 2021) (Fig. 1). Study materials were distributed from community centers or were delivered at home. Due to the limited amount of available GPS loggers and accelerometers, a maximum of 50 individuals participated in the same week. After six days of data collection, the study materials were picked up by a researcher at participants’ home. In each round of data collection, participants were measured during the same time of the year, to minimize the effects of seasons and the amount of daylight on the results. During the second follow-up, measurements were paused between the 18th of December 2020 and 1st of April 2021 to comply with COVID-19 regulations. The experiment was registered at the Netherlands Trial Register (NL8108, registered at 23/10/2019). After review of the study protocol, the medical ethical committee of the Maastricht University Medical Center (MUMC+) decided that formal ethical approval was not required (METC 16-4-109).

### Measurements

#### Sociodemographic characteristics

Age, gender, educational level, work status and car ownership were assessed using a questionnaire. Gender was dichotomized in male (0) and female (1). Educational level was assessed following the ISCED 2011 guidelines [23]. To create equal sized groups, participants with basic and intermediate levels of education were merged into the lower educated group (0) and individuals with a degree at a university of applied sciences or higher were merged in the higher educated group (1). Work status (0 = not employed, 1 = employed) and car ownership (0 = no car in household, 1 = one or more cars in household) were also dichotomized to create dummy variables for further analyses.

#### Health-related quality of life, social activities and meaningfulness

Health-related quality of life was measured using the EQ-5D-3 L questionnaire, which assesses five domains of health (mobility, self-care, daily activities, pain and mood) on three levels (no problems, any problems, severe problem) [24]. Due to the relatively healthy sample, scores on the EQ-5D-3 L were high for most participants. Some participants scored ‘any problems’ and almost none of the participants reported ‘severe problems’ on any of the domains. To deal with this homogeneity, the categories of ‘any problems’ and ‘severe problems’ were merged, and scores were coded as 0 (experiencing no problems) and 1 (experiencing any problems). Further, the total score was calculated based on the country-specific value sets that are available for this questionnaire [25]. Two additional subscales were added to evaluate social activities and meaningfulness. The subscales consisted of items that were scored on a five-point scale ranging from totally disagree to totally agree. For social activities, two items were included, 1) ‘*I regularly participate in activities in my neighborhood*’, and 2) ‘*I have many friends/acquaintances in my neighborhood*’. For the subscale meaningfulness, three items were included, 1) ‘*I feel in control of my life*’, 2) ‘*I have a future perspective in my life*’, and 3) ‘*I pursue goals and ideals in my life*’. For both subscales, total scores were calculated by summing the scores on the individual items. The internal validity of the subscales was acceptable to good (α = 0.613 and α = 0.837, for social activities and meaningfulness, respectively).

#### Physical activity, sedentary behavior and active transport

Physical activity, sedentary behavior and active transport were assessed using an accelerometer (Actigraph GT3X+) and GPS-logger (Qstarz BTQ1000XT). The devices were worn for 6 consecutive days using an elastic band on the hip. The devices had to be taken off at night to charge the battery of the GPS-logger, and during water activities (i.e. showering, swimming) and contact sports to prevent damage.

The accelerometer recorded data with epochs of 10s and the GPS-logger with epochs of 15 s. HABITUS was used to filter, convert and merge the datasets into 60s epochs (www.habitus.eu). Freedson 1998 cut off points were used to distinguish between sedentary behavior (SB; 0-100 counts per minutes (cpm)), light intensity physical activity (LPA) and moderate-to-vigorous intensity physical activity (MVPA) [26]. Periods of more than 60 min of 0 cpm were regarded as non-wear time. Datapoints were marked as being part of a trip or being stationary based on the speed and distance between two consecutive epochs. If the distance between two consecutive points was ≥ 100 m and the duration exceeded 120 s, data were marked as a trip. A stop of at least 120 s at one location was marked as a pause point and a pause of more than 180 s was marked as the endpoint of a trip. The trip detection algorithm had an accuracy of 92.5% [27]. Datapoints that were part of a trip were selected to determine transport-based SB, LPA and MVPA. Outcome measures on transport-based LPA, MVPA and SB were presented as the percentage of the total time spent in transport. Both trip and non-trip datapoints were selected to determine total PA levels. Similarly, LPA, MVPA and SB were presented as the percentage of the total measurement time that was spent in each category.

### Statistical analyses

All statistical analyses were performed in SPSS version 27 (IBM Corp., Armonk, NY, USA). Descriptive statistics were used to describe sample characteristics. T-tests and chi-square tests were performed to explore between-group differences in all covariates. T-tests and one-way ANOVA were used to examine between-group differences in outcome measures. Paired-samples T-tests were used to test within-group differences between baseline and follow-up. To test intervention effects, we used linear mixed models. This type of statistical models accounts for repeated measures within individuals, and is able to handle missing data in a longitudinal sample, using the values of covariates at baseline. For each outcome measure, a model was composed. Time was entered as a fixed factor and we accounted for repeated measures within persons. Each model was adjusted for age, gender, educational level, work status, and car-ownership. The models were supplemented with an interaction term between time x area-based exposure group to explore intervention effects. For all statistical analyses, a p-value of 0.05 was used as threshold for statistical significance.

## Results

At baseline 757 participants were recruited of which 642 provided valid accelerometer and questionnaire data. Of these 642 participants, 362 provided valid data at T0 and T1, and finally 241 provided valid data at T0, T1 and T2, which corresponds to a response rate of 38% at follow-up II. Sensitivity analyses showed that drop-outs were significantly younger (t = 3.624, p < .001) and less often a car owner (X^2^ = 7.648, p = .006).

### Description of the sample

Of all 241 participants, 105 were part of the maximal exposure group, 80 of the minimal exposure group and 56 participants we part of the no exposure group 7 (Table 2). The mean age of the sample was 59.8 years (SD = 12.8). The mean age of the minimal exposure groups was significantly higher than the mean age of the maximal exposure group (t = 2.367, p = .019) and the no exposure group (t = 2.429, p = .016). About half of the sample was male and about half of the sample was employed (45.9%). In total, 90.5% of the sample had access to at least one car in their household. No association was found between the exposure group and gender (X^2^ = 1.752, p = .417), educational level (X^2^ = 3.869, p = .145) work status (X^2^ = 2.981, p = .225) or car ownership (X^2^ = 0.410, p = .815). The mean minutes of SB, LPA and MVPA were comparable across the groups. The mean minutes per day of transport-based SB was significantly lower in the no exposure group compared to the minimal exposure group (t=-2.619, p = .010), but not compared to the maximal exposure group. The average baseline score on health-related quality of life was 0.92 for the total sample and did not differ between exposure groups. For social activities, the score for the maximal exposure group was significantly lower than for the no exposure group. Finally, the baseline score on meaningfulness was 12.2, and no differences between the groups were observed.

**Table 2:** Baseline characteristics of longitudinal sample

	Total sample (N = 241)	Maximal exposure (N = 105)	Minimal exposure (N = 80)	No exposure (N = 56)
**Sociodemographic characteristics**				
Age (mean (SD))	59.8 (12.8)	58.5 (12.6)	62.6 (12.0)*	57.6 (13.9)
Gender (% male)	52.3	58.1	46.3	50.0
Educational level (% higher educated)	53.7	50.5	49.4	66.1
Work status (% working)	45.9	48.6	38.3	51.8
Car ownership (% having ≥ 1 car in household)	90.5	88.6	91.4	92.9
**Physical activity and sedentary behavior**				
SB (mean min/day (SD))	552.4 (86.9)	544.1 (91.1)	561.7 (93.6)	554.3 (86.9)
LPA (mean min/day (SD))	268.3 (66.5)	267.6 (64.9)	267.3 (70.9)	271.0 (63.9)
MVPA (mean min/day (SD))	33.4 (22.5)	34.3 (23.1)	33.1 (24.0)	32.0 (19.2)
Transport-based SB (mean min/day (SD))	78.5 (43.3)	77.8 (41.1)	87.2 (46.1)	67.1 (41.2)†
Transport-based LPA (mean min/day (SD))	51.1 (21.3)	49.6 (19.3)	55.6 (23.9)	47.4 (19.9)
Transport-based MVPA (mean min/day (SD))	22.2 (18.6)	22.8 (19.3)	21.4 (18.8)	22.2 (16.9)
**Health-related quality of life**				
Health-related quality of life (mean (SD))	0.92 (0.12)	0.94 (0.11)	0.93 (0.11)	0.93 (0.13)
Social activities (mean (SD))	6.05 (1.83)	5.80 (1.97)^α^	6.05 (1.67)	6.49 (1.73)
Meaningfulness (mean (SD))	12.2 (1.90)	12.1 (2.22)	12.2 (1.69)	12.3 (1.52)

SD = standard deviation; SB = sedentary behavior; LPA = light physical activity; MVPA = moderate-to-vigorous physical activity; * = significantly different from age in maximal and no exposure groups; †= significantly different from transport-based SB in minimal exposure group; α = significantly different from social activities in no exposure group

### Physical activity, sedentary behavior and active transport

The average total weartime of the accelerometer and GPS logger was about 14 h per day and did not significantly change between baseline and follow-up (Table 3). Baseline levels of (transport-based) sedentary behavior and physical activity did not differ between groups. For the maximal exposure group, the percentage of total and transport-based physical activity levels and SB did not significantly change over time. In absolute terms, the amount of transport-based SB decreased with 11.58 min (t=-2.728, p = .007). In the minimal exposure group, transport-based SB significantly decreased over time, while transport-based MVPA increased over time. These changes corresponded with a decrease of 13.36 min of transport-based SB (t=-2.510, p = .014) and an increase of 5.04 min of transport-based MVPA (t = 2.072, p = .042). For the no exposure group, an inverse trend was visible: the total percentage SB increased significantly, while total LPA and transport-based LPA decreased over time. In absolute terms, total LPA decreased with 28 min per day (t=-5.777, p < .001).

**Table 3:** Mean total and transport-based physical activity levels

		Baseline	Follow-up II	t, p
**Weartime**	Hours per day (Mean (SD))	14.23 (1.32)	14.08 (1.28)	1.74, 0.083
**Maximal exposure** **(N = 105)**	% SB	64.17 (7.86)	64.44 (8.54)	0.42, 0.679
	% LPA	31.79 (7.65)	31.57 (7.86)	-0.32, 0.749
	% MVPA	4.03 (2.66)	3.99 (2.74)	-0.19, 0.85
	% transport-based SB	50.44 (13.86)	47.63 (13.28)	-1.87, 0.065
	% transport-based LPA	34.04 (10.02)	34.57 (11.38)	0.41, 0.679
	% transport-based MVPA	15.51 (11.91)	17.80 (12.89)	1.72, 0.088
**Minimal exposure** **(N = 80)**	% SB	65.04 (8.70)	65.90 (10.06)	1.03, 0.305
	% LPA	31.12 (8.32)	30.17 (9.58)	-1.19, 0.237
	% MVPA	3.85 (2.79)	3.93 (2.97)	0.26, 0.795
	% transport-based SB	**51.37 (13.35)**	**47.77 (13.14)**	**-2.13, 0.037**
	% transport-based LPA	35.04 (12.22)	35.37 (12.12)	0.21, 0.836
	% transport-based MVPA	**13.57 (11.44)**	**16.86 (13.13)**	**2.34, 0.022**
**No exposure** **(N = 56)**	% SB	**64.81 (6.10)**	**67.01 (7.58)**	**2.21, 0.031**
	% LPA	**31.46 (5.99)**	**29.10 (7.19)**	**-2.49, 0.016**
	% MVPA	3.73 (2.21)	3.89 (3.08)	0.40, 0.689
	% transport-based SB	46.68 (14.85)	50.16 (15.48)	1.54, 0.128
	% transport-based LPA	**35.57 (12.00)**	**31.18 (11.76)**	**-2.44, 0.018**
	% transport-based MVPA	17.74 (13.87)	18.67 (15.48)	0.40, 0.691

LPA = light physical activity; MVPA = moderate-to-vigorous physical activity; SB = sedentary behavior; SD = standard deviation

### Intervention effects on transport-based and total physical activity levels

**Figure 2:**
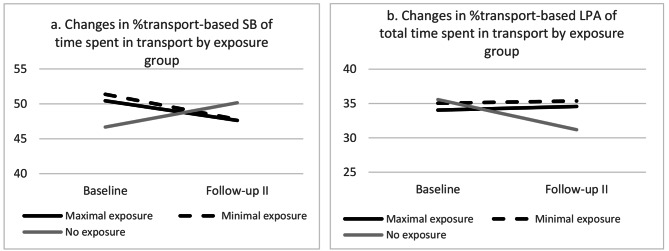
**a-b**. Visualization of significant interaction terms between exposure group and transport-based SB (left) and transport-base LPA (right ). SB = sedentary behavior; PA = physical activity

The interactions between time x exposure group were examined to explore whether changes over time were different for the three exposure groups. Significant interaction effects were found for both the minimal exposure and maximal exposure group, indicating that changes over time were significantly different for the no exposure group. In both exposure groups, the percentage transport-based SB was significantly lower and the percentage transport-based LPA was significantly higher compared to the no exposure group (Table 4). The significant interaction terms for transport-based SB and transport-based LPA are visualized in Fig. 2a and b.

**Table 4:** Intervention effect for exposure groups and users, on total and transport-based physical activity levels

Adjusted model (age, gender, educational level, employment, car ownership)				
	Follow-up II vs. Baseline			
	Maximal vs. No exposure		Minimal vs. No exposure	
Total PA	*B (95% CI)*	*p*	*B (95% CI)*	*p*
% SB	-1.91 (-4.20; 0.38)	0.102	-1.63 (-4.05; 0.80)	0.188
% LPA	1.90 (-0.38; 4.18)	0.103	1.68 (-0.73; 4.10)	0.171
% MVPA	-0.03 (-0.93; 0.87)	0.950	-0.08 (-1.03; 0.87)	0.865
Transport-based PA				
% SB	**-6.25 (-11.38; -1.13)**	**0.017**	**-7.52 (-12.92; -2.12)**	**0.007**
% LPA	**4.92 (0.54; 9.30)**	**0.028**	**4.77 (0.15; 9.38)**	**0.043**
% MVPA	1.36 (-3.30; 6.02)	0.566	2.81 (-2.09; 7.72)	0.260

β = beta coefficient; 95%CI = 95 confidence interval; p = p-value; LPA = light physical activity; MVPA = moderate-to-vigorous physical activity; SB = sedentary behavior

### Health-related quality of life, social activities and meaningfulness

In all three groups, the trend of the total score for health-related quality of life was negative, implying a decline of well-being, but no significant changes were found for the maximal and no exposure groups (Table 5). For the minimal exposure group, the total score on the health-related quality of life decreased significantly between T0 and T2 (t=-2.09, p = .039).The score for social activities at baseline was significantly lower in the maximal exposure group, compared to the no exposure group. Albeit not significant, the trends for social activities and meaningfulness were positive for the maximal exposure group and negative for the minimal and no exposure group. One-way ANOVA analyses showed no between-group differences at follow-up-II.

**Table 5:** Changes in health-related quality of life, social activities and meaningfulness

	Baseline	Follow-up II	t, *p* (T2-T0)
**Maximal exposure (N = 105)**			
Health-related quality of life	0.94 (0.11)	0.89 (0.18)	-1.72, 0.089
Social activities	5.80 (1.97)*	5.91 (1.83)	0.71, 0.479
Meaningfulness	12.07 (2.22)	12.24 (2.02)	0.82, 0.416
**Minimal exposure (N = 80)**			
Health-related quality of life	**0.93 (0.11)**	**0.91 (0.11)**	**-2.09, 0.039**
Social activities	6.05 (1.67)	5.94 (1.66)	-0.60, 0.548
Meaningfulness	12.21 (1.69)	11.86 (1.81)	-1.76, 0.082
**No exposure (N = 56)**			
Health-related quality of life	0.93 (0.13)	0.89 (0.14)	-1.75, 0.084
Social activities	6.49 (1.73)	6.19 (1.78)	-1.55, 0.125
Meaningfulness	12.30 (1.52)	11.97 (1.56)	-1.67, 0.100

*= score at baseline significantly different from the no exposure group; p = p-value; t = t-value

### Intervention effects on health-related quality of life, social activities and meaningfulness

**Fig. 3:**
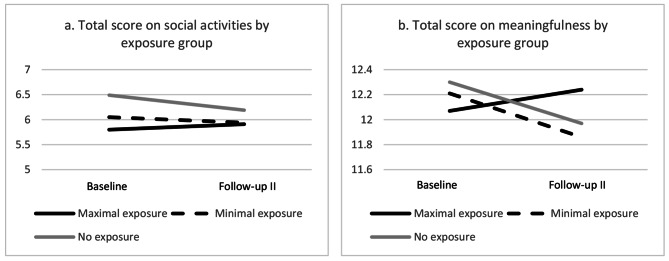
**a-b.** Visualization of significant interaction terms between exposure group and total score on social activities (left), and between exposure group and total score on meaningfulness (right)

Beta’s of the interactions between time x exposure group showed an increase of the score on social activities and meaningfulness in the maximal exposure group compared to the no exposure group, while this was not the case in the minimal exposure group. However, these interactions were not statistical significant (Table 6). The interactions are also visualized in Fig. 3a-b.

**Table 6:** Intervention effect for exposure groups on health-related quality of life, social activities and meaningfulness

	Follow-up II vs. Baseline			
	Maximal vs. No exposure		Minimal vs. No exposure	
	*B (95% CI)*	*p*	*B (95% CI)*	*p*
Health-related quality of life	0.004 (-0.04, 0.049)	0.862	0.006 (-0.04, 0.05)	0.793
Social activities	0.45 (-0.09, 0.99)	0.101	0.27 (-0.30, 0.83)	0.352
Meaningfulness	0.44 (-0.14, 1.02)	0.134	-0.01 (-0.62, 0.60)	0.982

β = beta coefficient; 95%CI = 95 confidence interval; p = p-value

## Discussion

### Main findings

The aim of this study was to assess the effects of a major urban redesign project, on physical activity levels, active transport and health-related quality of life in adults, at 29–39 months after opening of the new infrastructure. Despite the COVID-19 outbreak and lockdown policies during follow-up, both the maximal and minimal exposure groups showed significantly different trends regarding transport-based physical activity levels compared to the no exposure group; In the exposure groups, the trend for transport-based SB was negative, while it was positive for the no exposure group. Also, transport-based LPA remained stable in both exposure groups, while it significantly decreased in the no exposure group. At this point in time, no significant intervention effects were found for total daily physical activity levels. Also, although the health-related quality of life outcomes increased in the maximal exposure group compared to the minimal and no exposure groups, these effects were not statistically significant.

### Physical activity, sedentary behavior and active transport

Previous research showed promising results regarding the effects of infrastructural changes on physical activity and active transport [4, 5]. However, especially in larger infrastructural projects, effects are generally small or non-existing [9]. Large changes in entire systems may lead to changes in physical activity, active transport and sedentary behavior, but also to compensatory adaptive processes and feedback loops that make it harder to assess clear mechanistic pathways and direct effects [28]. Most of the available evaluations of infrastructural projects have follow-up times up to 24 months which is, according to more recent findings, a rather short term to detect behavioral changes in physical activity and active transport. The assumption that infrastructural interventions might need up to 3 years to result in population-level changes in (transport-based) physical activity is confirmed by the current study. The short-term evaluation of this project found favorable intervention effects on transport-based sedentary behavior [28], but the effect sizes were relatively small. The current study revealed that these trends were sustained over time, and effect sizes almost doubled at the second follow-up. This confirms that rigorous changes in the built environment can lead to sustainable behavioral change, but changes take more time to occur and to be measurable [19]. Therefore, it is important to ensure long-term follow-ups when evaluating large-scale built environmental interventions, to explore the full potential of the newly designed areas.

The recommendations of recent systematic reviews and research to prolong follow-up times turned out to be valid, but also has some challenges. In research in general, drop-outs are a threat to research designs and various strategies are followed to prevent loss to follow-up. In studies in which place matters, loss to follow-up is not only a result of the loss of interest or due to personal circumstances of participants, but also a result of people that are moving. For example, in the intervention area lives a relatively large population of students, which are moving more often than the general population. This could also be the explanation for the finding that drop-outs between baseline and follow-up were younger and less often a car owners. Also, the COVID-19 contact restricting measures could have caused additional drop-outs, especially for more vulnerable individuals. Future research should investigate how measurement methods, incentives and other measures can improve the retention of individuals in longer-term evaluations.

Not only the relative amount of transport-based physical activity decreased, but the absolute amount of transport-based sedentary behavior decreased as well. In the maximum and minimum exposure groups transport-based sedentary behavior decreased with 11 and 13 min per day, respectively. In addition, trends of transport-based light physical activity were significantly different for the exposure groups compared to the no exposure groups, as transport-based LPA decreased over time for the no exposure group while it remained stable for both exposure groups. Previous research found that in some contexts, active transport accounted for 31% of the total energy expenditure and for 13% of the sedentary time during 7 measurement days [29]. But despite the changes in transport-based physical activity levels, we found no changes on the total physical activity levels. Possibly, the effects on transport-based physical activity are still too small to result in changes in the total physical activity levels. This could be due to the small scale of Dutch cities, which make trip distances in the Netherlands rather short [30], especially in comparison with countries in the Anglosphere such as the UK and the USA [31]. This in turn minimizes the effects of active transport trips on the total amount of physical activity. Also, it is possible that the increase in transport-based physical activity is compensated by less physical activity in other domains, but this was not evaluated in the current study [32]. Lastly, there might be a ceiling effect, as the Netherlands has already a large mode share of cycling, due to its high level of cycling infrastructure [33].

Remarkably, the changes in transport-based physical activity were comparable for the minimal and maximal exposure group, with a slightly larger effect for the minimal exposure group. This could be explained by the improved connectivity in the maximal exposure group after the tunneling of the highway. While previously, only a few intersections were available for pedestrians and cyclists, it is now possible to cross the Green Carpet at various points. Trips can now be more efficient and thus shorter. However, an in-depth trip-analysis is necessary to further investigate this argument. Further, for the minimal exposure group, the Green Carpet might act as a new destination or attractive route for active transportation. Also, in the six years between baseline and follow-up, some smaller investments in the built environment of the minimal exposure group were made, such as a new ‘slow traffic’ route, which aimed to improve the livability and creating greater connectivity in the residential area of this group. This might have caused a shift regarding walking and bicycling for transportation.

### Health-related quality of life, social activities and meaningfulness

Both social activities and meaningfulness showed a positive trend over time for the maximal exposure group, while these were negative for the minimal and no exposure group. Although these differences were not statistically significant, the maximal exposure group is following a different trend after the opening of the Green Carpet. The lack of statistical significance might be caused by the relatively small sample size.

The score on health-related quality of life showed a slightly negative trend over time for all groups, but this decrease was only significant for the minimal exposure group. The negative trend of the health-related quality of life score might accelerate as age increases, as the minimal exposure group was significantly older and showed a significant decline in health-related quality of life score over time. In an evaluation of a major infrastructural intervention in Belfast, health-related quality of life scores also followed a negative trend, with significant decrease over time [34]. However, this study found a significantly smaller decline in the intervention group [34]. Furthermore, the second follow-up took place during the COVID-19 pandemic. Previous research showed that the pandemic and its restrictions in movement and social contacts had a negative impact on quality of life [35, 36], which could explain the decline in health-related quality of life as well.

According to socioecological models, health and wellbeing are influenced by proximate factors at the micro/interpersonal level such as health behaviors [37]. This means that changes in the built environment should change proximate factors such as infrastructural stressors (e.g. environmental conditions and safety), health behaviors or social participation to affect general health and well-being. Therefore, it might even take more time before effects in health and wellbeing are present and measurable. To improve overall health and wellbeing, or to prevent further declines, larger changes in proximate factors such as the total amount of physical activity might be necessary.

### Strengths and limitations

The results of this study have to be interpret in light of the challenges and limitations that come with natural experiments as research design. First, it is practically and ethically impossible to randomly assign participants to intervention and control groups. Also, it is not possible to control for all contextual factors that might be relevant when evaluating interventions in a real-life setting. However, even though natural experiments have considerable methodological differences with for example randomized controlled trials, they lead to much needed evidence regarding population-level strategies to improve behavior and health [18]. Some of the contextual factors that might have influenced our results are discussed below.

The follow-up measurement took place between September 2020 and July 2021, which was in the middle of the COVID-19 pandemic. During this period, several contact limiting measures were in place to reduce the spread of the virus. Also, all inhabitants of the Netherlands were encouraged to work from home as much as possible, which has affected the amount of commuting. Although no large changes in the total amount of transport was observed in this study, the mode share has probably changed over time. Research from the Netherlands Mobility Panel has shown that since the COVID-19 pandemic, people are more negative about the use of public transportation, and more positive about trips by car [38]. At the time of the total lockdown and curfew (between the 18th of December 2020 and 1st of April 2021), the measurements were paused to comply with COVID-19 regulations and to limit the effects of the measures on the results of this study. However, while it is impossible to quantify the effects of the preventive measures on all outcomes, it is very likely that the pandemic has had some effects on the results. As all COVID-19 measures were implemented country-wide, the effects are expected to be similar in the exposure and no exposure groups which may cancel out the influence of these measures when investigating the trends over time between the exposure and no exposure groups.

Apart from the COVID-19 pandemic, other contextual factors might have influenced the outcomes during the six years between baseline and follow-up, as the Green Carpet is not a stand-alone intervention. The Green Carpet project changed the connectivity, amount of traffic an aesthetics of the affected neighborhoods. But there also might be a change in the social environment of these neighborhoods that comes along with new infrastructure, new dwellings and new inhabitants [39]. Possible gentrification and psychological displacement of the individuals that remained living in the study area can also have an effect on the mental health status of these individuals [40]. Qualitative research is needed to further investigate the effects contextual factors and the social environment on the results of this evaluation. Further, the sample size of this longitudinal analysis was relatively small, as the dropout rate increased over time. Finally, 38% of the participants at baseline provided valid data at baseline and follow-up. As a result, some of the non-significant findings might be due to a lack of power. Lastly, when interpreting the results of this study, one should take into account that the study sample was higher educated and older compared to the general Dutch adult population [41, 42].

## Conclusion

This study identified significant intervention effects of a major urban redesign project on transport-based physical activity levels at 29–39 months after opening of the newly designed area. The results showed significant intervention effects on transport-based sedentary behavior and transport-based light intensity physical activity for both exposure groups, compared to the no exposure group. In comparison to the shorter-term evaluation and despite the COVID-19 physical isolation policies, the effect sizes increased over time. These results emphasize the potential of the built environment in changing and sustaining healthy behavior over a longer period of time. Scores on social activities and meaningfulness increased in the maximal exposure group while it decreased in the minimal and no exposure groups, but changes over time were not statistically significant. The results of this study should be interpret in light of the limitations and challenges that come with the design of a natural experiment. Finally, the intervention area is still under construction, so even longer follow-up terms might be needed to explore its full potential.

## Electronic supplementary material

Below is the link to the electronic supplementary material.

Supplementary Material 1

## Data Availability

The datasets generated during and/or analyzed during the current study are available from the corresponding author on reasonable request. Competing interests. The authors declare that they have no competing interests.

## Abbreviations

GPS: global positioning system
HABITUS: Human Activity Behavior Identification Tool and data Unification System
HRQoL: health-related quality of life
LPA: light intensive physical activity
MVPA: moderate-to-vigorous physical activity
PA: physical activity
SA: social activities
SB: sedentary behavior
SD: standard deviation
