# Supplementary material for: Effects of major urban redesign on sedentary behavior, physical activity, active transport and health-related quality of life in adults
Source: BMC Public Health. 2023 Jun 15;23:1157. doi: 10.1186/s12889-023-16035-6 (PMC10267553; doi:10.1186/s12889-023-16035-6)
Supplement: Supplementary file 1 — Supplementary Material 1 [file 12889_2023_16035_MOESM1_ESM.docx]

# Supplementary material


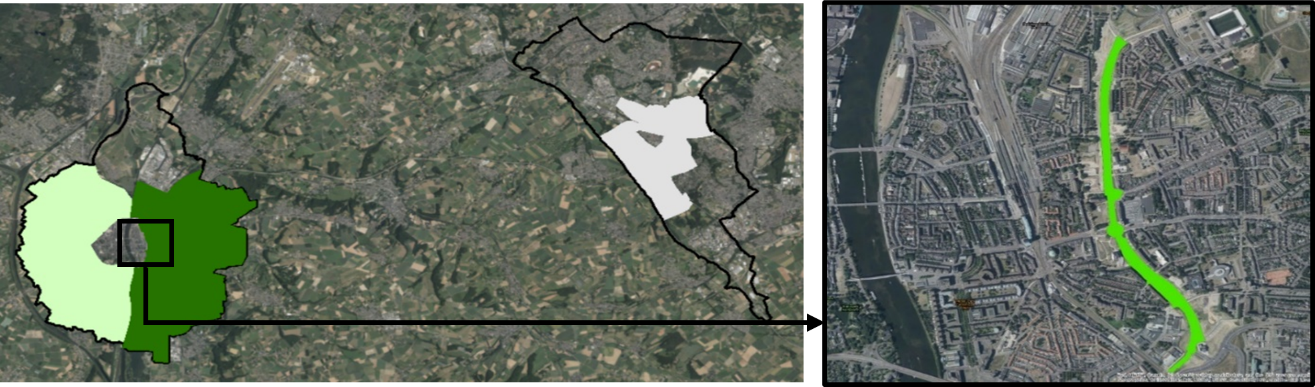


Figure S1. Three area-based exposure groups. Dark green: maximal exposure group in East Maastricht; Light green: minimal exposure group in West Maastricht; White: no exposure group in Heerlen. Cutout, light green: Green Carpet.
